# Idiopathic retroperitoneal fibrosis diagnosed by endoscopic ultrasonography‐guided fine‐needle biopsy

**DOI:** 10.1002/jgh3.12431

**Published:** 2020-10-14

**Authors:** Yujiro Kawakami, Yumemi Takada, Keisuke Ishigami, Takehiro Hirano, Kohei Wagatsuma, Yoshiharu Masaki, Ayako Murota, Masayo Motoya, Mitsuhiro Tsujiwaki, Hiroki Takahashi, Hiroshi Nakase

**Affiliations:** ^1^ Department of Gastroenterology and Hepatology Sapporo Medical University School of Medicine Sapporo Japan; ^2^ Department of Surgical Pathology Sapporo Medical University School of Medicine Sapporo Japan; ^3^ Department of Rheumatology and Clinical Immunology Sapporo Medical University School of Medicine Sapporo Japan

**Keywords:** endoscopic ultrasonography‐guided fine needle biopsy, IgG4‐related disease, retroperitoneal fibrosis

## Abstract

We demonstrate a case, in which endoscopic ultrasonography‐guided fine‐needle biopsy (EUS‐FNB) was useful for determining the diagnosis of lesions of retroperitoneal fibrosis. In our case, accessing the retroperitoneal lesions by conventional percutaneous biopsy procedures was not feasible due to the difficulty of avoiding the inferior vena cava and ureter. We believe that our case demonstrates a unique approach for performing histological analysis in a challenging case.

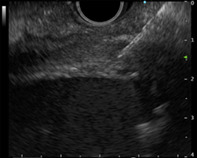

A 67‐year‐old man presented with back pain. Computed tomography (CT) revealed right hydronephrosis caused by a soft‐tissue mass surrounding the abdominal aorta and bilateral common iliac arteries (Fig. 1a). Positron‐emission tomography examination revealed that the lesion was localized at the retroperitoneum (Fig. 1b). Blood tests revealed elevated creatinine (1.4 mg/dL) and mild elevation of IgG4 (140 mg/dL). We could not perform CT‐guided percutaneous needle biopsy of the retroperitoneal lesions because of the difficulty in avoiding the inferior vena cava and ureter during the procedure. To diagnose the etiology, we performed endoscopic ultrasonography‐guided fine‐needle biopsy (EUS‐FNB). A hypoechogenic tissue formation surrounding the descending aorta was visualized via scanning from the descending part of the duodenum, and the lesion was punctured with a 22‐gauge Franseen needle (Acquire, Boston Scientific Corp., Natick, MA, USA) under EUS guidance (Fig. 1c). Histopathology revealed an inflammatory infiltration with glass‐like fibrous tissue, no malignant lesion, and 10% of the IgG4/IgG plasma cell ratio (Fig. 1d–f). Finally, based on the pathological findings, we diagnosed the patient with idiopathic retroperitoneal fibrosis (RPF). However, the patient did not meet the diagnostic criteria for IgG4‐related RPF.^1^ The patient received treatment with oral prednisolone, and the lesion was dramatically reduced (Fig. 1g).

**Figure 1:**
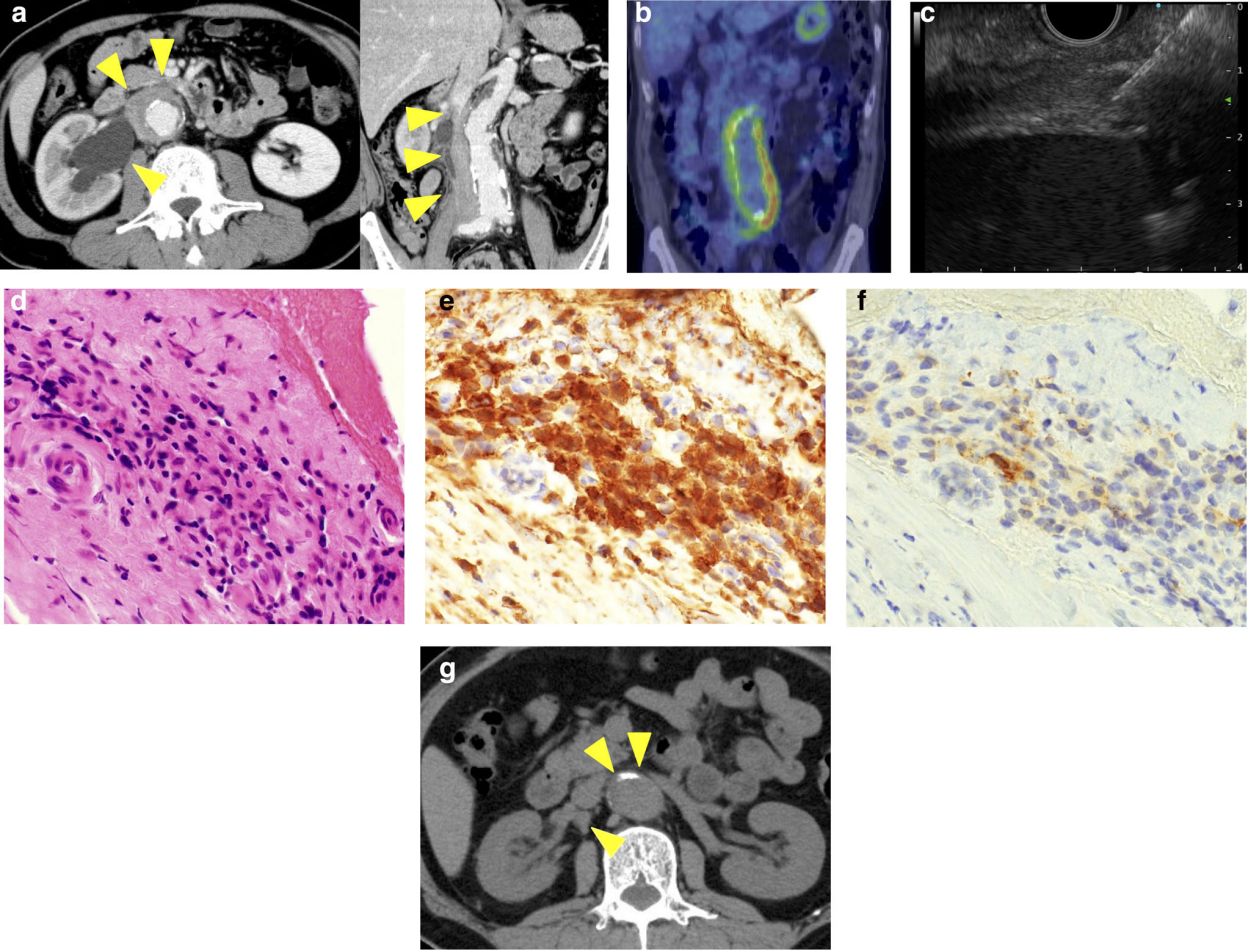
(a) Computed tomography (CT) revealed right hydronephrosis caused by a soft‐tissue mass surrounding the abdominal aorta and bilateral common iliac arteries (arrow heads). (b) Positron‐emission tomography examination revealed that the lesion was localized at the retroperitoneum. (c) Endoscopic ultrasonography‐guided fine‐needle biopsy (EUS‐FNB) was performed. (d, e, f) Histopathology revealed an inflammatory infiltration with glass‐like fibrous tissue, no malignant lesion, and 10% of the IgG4/IgG plasma cell ratio (d, hematoxylin stain ×600; e, IgG stain ×600; f, IgG4 stain ×600). (g) After treatment with oral prednisolone, the lesion was reduced.

RPF is a rare disease characterized by the presence of a fibroinflammatory tissue, which usually surrounds the abdominal aorta and the iliac arteries and extends into the retroperitoneum to envelop neighboring structures, such as the ureters.^2^ RPF can be divided into idiopathic and secondary subsets, which can be caused by drugs, malignant diseases, and infections.^3^ In addition, the etiology of idiopathic RPF includes IgG4‐related disease or non‐IgG4‐related disease.^3^ Therefore, histological analysis is important to evaluate idiopathic (IgG4 or non‐IgG4‐related) and secondary forms of RPF.^4^ Retroperitoneal biopsy is usually performed via open, laparoscopic, and CT‐guided approaches.^5^ CT‐guided percutaneous needle biopsy of the retroperitoneal lesion is less invasive and is the preferred choice but is considered technically challenging because of the extensive depth of the lesions and their proximity to vital structures such as vessels and ureters.^6^ A recent study demonstrated that EUS‐FNB was useful for the histological diagnosis of type 1 autoimmune pancreatitis in IgG4‐related disease.^7^ Furthermore, the latest prospective study on the diagnosis of type 1 autoimmune pancreatitis demonstrated that the diagnostic accuracy of 22‐gauge Franseen needles was higher than that of 20‐gauge forward‐bevel needles.^8^ If the CT‐guided percutaneous biopsy procedure for pathological diagnosis is not feasible, as in the current case, EUS‐FNB should be considered.
